# Changes of ribosomal protein S3 immunoreactivity and its new expression in microglia in the mice hippocampus after lipopolysaccharide treatment

**DOI:** 10.1186/cc10909

**Published:** 2012-03-20

**Authors:** JH Cho, CW Park, HY Lee, MH Won

**Affiliations:** 1 Kangwon National University, Chuncheonsi, South Korea

## Introduction

Lipopolysaccharide (LPS) has been commonly used as a reagent for a model of systemic inflammatory response. Ribosomal protein S3 (rpS3) is a multifunctional protein that is involved in transcription, metastasis, DNA repair and apoptosis. In the present study, we examined the changes of rpS3 immunoreactivity in the mouse hippocampus after systemic administration of 1 mg/kg LPS.

## Methods

Six-week-old male ICR mice were purchased from the Jackson Laboratory (Bar Harbor, ME, USA). LPS (Sigma, St Louis, MO, USA) was dissolved in saline, and administered intraperitoneally with 1.0 mg/kg/10 ml dose. The control animals were injected with the same volume of saline. Mice (*n *= 7 at each time point) were sacrificed at designated times (3, 6, 12, 24, 48 and 96 hours after LPS treatment). The brain tissues were cryoprotected by infiltration with 30% sucrose overnight. Thereafter, frozen tissues were serially sectioned on a cryostat (Leica, Wetzlar, Germany) into 30-μm coronal sections, and they were then collected into six-well plates containing 0.1 M PBS.

## Results

From 6 hours after LPS treatment, rpS3 immunoreactivity was decreased in pyramidale cells of the hippocampus proper and granule cells of the dentate gyrus. At this point in time, rpS3 immunoreactivity began to increase in nonpyramidal cells and nongranule cells in the hippocampus. From 1 day after LPS treatment, rpS3 immunoreactivity in pyramidal and granule cells was hardly detected, and nonpyramidal and nongranule cells showed strong rpS3 immunoreactivity. Based on double immunofluorescence staining, microglia, not astrocytes, expressed strong rpS3 immunoreactivity at 1 and 2 days after LPS treatment.

## Conclusion

These results indicate that changes in rpS3 immunoreactivity in pyramidal and granule cells and rpS3 expression in activated microglia after LPS treatment may be associated with the neuroinflammatory responses in the brain.

